# Biofabricated Fatty Acids-Capped Silver Nanoparticles as Potential Antibacterial, Antifungal, Antibiofilm and Anticancer Agents

**DOI:** 10.3390/ph14020139

**Published:** 2021-02-09

**Authors:** Mohammad Azam Ansari, Sarah Mousa Maadi Asiri, Mohammad A. Alzohairy, Mohammad N. Alomary, Ahmad Almatroudi, Firdos Alam Khan

**Affiliations:** 1 Department of Epidemic Disease Research, Institute for Research and Medical Consultations (IRMC), Imam Abdulrahman Bin Faisal University, P.O. Box 1982, Dammam 31441, Saudi Arabia; 2 Department of Biophysics, Institute for Research and Medical Consultations (IRMC), Imam Abdulrahman Bin Faisal University, Dammam 31441, Saudi Arabia; smasiri@iau.edu.sa; 3 Department of Clinical Laboratory Sciences, College of Applied Medical Sciences, Qassim University, Qassim 51431, Saudi Arabia; dr.alzohairy@gmail.com; 4 National Center for Biotechnology, Life Science and Environmental Research Institute, King Abdulaziz City for Science and Technology, P.O. Box 6086, Riyadh 11451, Saudi Arabia; malomary@kacst.edu.sa; 5 Department of Stem Cell Biology, Institute for Research and Medical Consultations (IRMC), Imam Abdulrahman Bin Faisal University, P.O. Box 1982, Dammam 31441, Saudi Arabia; fakhan@iau.edu.sa

**Keywords:** metal nanoparticles, GC-MS, FT-IR, XRD, fatty acids, drug resistant, biofilm, colon cancer, polyherbal drug

## Abstract

The current study demonstrates the synthesis of fatty acids (FAs) capped silver nanoparticles (AgNPs) using aqueous poly-herbal drug Liv52 extract (PLE) as a reducing, dispersing and stabilizing agent. The NPs were characterized by various techniques and used to investigate their potent antibacterial, antibiofilm, antifungal and anticancer activities. GC-MS analysis of PLE shows a total of 37 peaks for a variety of bio-actives compounds. Amongst them, n-hexadecanoic acid (21.95%), linoleic acid (20.45%), oleic acid (18.01%) and stearic acid (13.99%) were found predominately and most likely acted as reducing, stabilizing and encapsulation FAs in LIV-AgNPs formation. FTIR analysis of LIV-AgNPs shows some other functional bio-actives like proteins, sugars and alkenes in the soft PLE corona. The zone of inhibition was 10.0 ± 2.2–18.5 ± 1.0 mm, 10.5 ± 2.5–22.5 ± 1.5 mm and 13.7 ± 1.0–16.5 ± 1.2 against *P. aeruginosa*, *S. aureus* and *C. albicans*, respectively. LIV-AgNPs inhibit biofilm formation in a dose-dependent manner i.e., 54.4 ± 3.1%—10.12 ± 2.3% (*S. aureus*)*,* 72.7 ± 2.2%–23.3 ± 5.2% (*P. aeruginosa*) and 85.4 ± 3.3%–25.6 ± 2.2% (*C. albicans*), and SEM analysis of treated planktonic cells and their biofilm biomass validated the fitness of LIV-AgNPs in future nanoantibiotics. In addition, as prepared FAs rich PLE capped AgNPs have also exhibited significant (*p* < 0.05 *) antiproliferative activity against cultured HCT-116 cells. Overall, this is a very first demonstration on employment of FAs rich PLE for the synthesis of highly dispersible, stable and uniform sized AgNPs and their antibacterial, antifungal, antibiofilm and anticancer efficacy.

## 1. Introduction

The growing pursuits in metal-based nanomaterials synthesis are hotly debated in several fields while acknowledging their unique physico-chemical and biomedical properties with specific advocacy for fitness in clinical settings as fascinating treatment modality, worldwide [1]. Considering that there is a wide scope to achieve desired properties in synthesized nanoparticles (NPs) including shape, size and stability by manipulating reaction conditions such as pH, temperature, concentration of metal precursors and concentration and nature of bio-reducing agents [2,3,4,5,6,7,8]. Besides, surface capping or encapsulation material of NPs deserves special importance due to being directly or indirectly concerned with limiting toxicity and cations release, affinities towards miscellaneous components of immediate environment, and cells surface, enhanced dispersity and prolonged stability and enhanced internalization propensity in several cells [5,9]. Owing to feasibility and proven antimicrobial properties, silver NPs (AgNPs) can be argued for being the most commonly synthesized and investigated nanostructure within metallic nanomaterial’s landscape, however, still yet holds consistent attention of researchers. Since ancient times, the Ag based formulations (e.g., metallic silver, silver nitrate and silver sulfadiazine) mediated clinical management of infections caused by bacteria, viruses and other eukaryotic microorganisms has been on top among metallic and metal oxides [10,11]. AgNPs have also been reported disrupting respiratory chain and cell division while releasing Ag^+^ in order to augment enhanced bacterial killing [12]. Recently, Zhao et al. [13] reported that coating of AgNPs can result in improved functionality and corrosion resistance of magnesium structures in biomedical settings. In this line, the study of Zhang et al. [14] underscored the stabilizing role of sodium alginate on calcium alginate nano-silver phosphate hybrid (CaAlg/nano-Ag_3_PO_4_) by demonstrating a clear-cut uniformity in Ag_3_PO_4_ NPs size (10–60 nm) and dispersity in CaAlg phase. Similarly, Labanni et al. [15] has revealed the three alkanolamine compounds viz. diethanolamine, monoethanolamine and triethanolamine could be used as a comprehensive capping/encapsulating medium to impregnate hydroxyapatite particles on AgNPs for antibacterial bone implants.

At one end, despite substantial increase in innovative technologies to restrain the emergence of multiple drugs resistance (MDR), there is no acclaimed antimicrobial agent to warrant the control of MDR crisis effectively. This has drifted the scientific interest towards nontraditional antimicrobial formulations to limit the MDR havoc timely. For instance, the integration of “nanoantibiotics” as new paradigms of antimicrobials in clinical settings including personnel protection equipment (e.g., gloves, masks and apron) and surgical devices were observed potentially effective to circumvent the drawbacks of traditional antibiotics [16,17,18]. To date, a plethora of nanomaterials, specifically metallic NPs exhibited elevated functionalities and safer administration in both, in vitro and in vivo when employed as nanoantibiotics to mitigate microbial infections [19,20,21]. In fact, advanced nanoantibiotics formulations can also be envisioned to offer several merits: (i) functional stability against enzymatic or pH degradation or inactivation, (ii) enhanced solubility and (iii) favorable biocompatibility [20]. Beyond, the co-delivery of non-toxic plant bio-actives adsorbed onto surface of bio-inspired NPs eventually enhance antimicrobial potential due to: (i) prolonged stability, (ii) greater dispersity in immediate environment, (iii) slow release of cations and (iv) enhanced intracellular uptake as compared to their bare surface NPs [22]. Nevertheless, taken the antimicrobial merits of AgNPs and low toxic herbal bio-actives together, a substantial interest of scientists in MDR field inclined to combine AgNPs bio-actives such as polyphenols, tannins, alkaloids, terpenoids, long and short chained hydrocarbons and proteins to ameliorate antimicrobial efficacy against Gram-positive and Gram-negative bacteria (e.g., *E. coli* and *S. aureus*) [5,22]. Hence, within the green chemistry premises, AgNPs have been fabricated with several green extracts obtained from an individual plant, animal tissue and microbial cell culture.

Precisely, Liv52 composition generally used as poly-herbal medicine (composed of *Mandur basma*, *Tamarix gallica* and herbal extracts of *Capparis spinosa*, *Cichorium intybus*, *Solanum nigrum*, *Terminalia arjuna* and *Achillea millefolium*) to prevent and control of cirrhosis, a chronic liver disease [23]. Thus, considering the bio-reducing ability of poly-herbal bio-actives in Liv52 extract (PLE), we have employed aqueous PLE for bio-reduction of Ag^+^ ions to Ag^0^ to obtained stable and highly monodispersed PLE capped AgNPs (LIV-AgNPs) in our one-pot synthetic scheme demonstrated in Figure 2. Besides, we also examined antimicrobial trends of LIV-AgNPs against MDR *Pseudomonas aeruginosa* (MDR-PA), methicillin-resistant *Staphylococcus aureus* (MRSA) and *Candida albicans* in planktonic as well as biofilm forms. In addition, the prepared LIV-AgNPs were also examined for their toxicity trend against cultured human colorectal carcinoma cell line (HCT-116 cells).

## 2. Results and Discussion

### 2.1. Physico-Chemical Characterization of LIV-AgNPs

#### 2.1.1. Synthesis and UV-Vis Analysis of LIV-AgNPs

**Figure 1:**
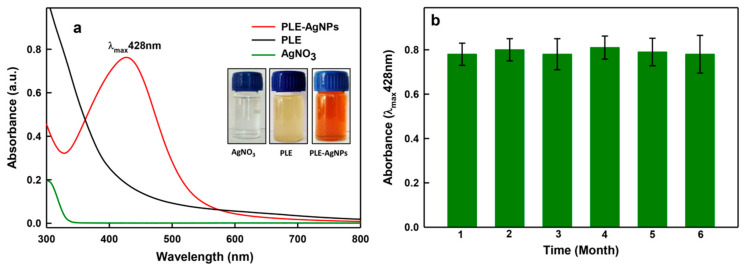
UV-Vis analysis of nanoparticles (NPs). Panel (**a**) demonstrate UV-Vis absorption peaks of pristine poly-herbal bio-actives in Liv52 extract (PLE), AgNO_3_ and LIV-AgNPs. Panel (**b**) shows stability of PLE capped silver NPs (LIV-AgNPs) based on surface plasmon resonance (SPR) measurements up to six months (error bars represent the mean ± SE of three replicates).

Briefly, an apparent color change in the reaction mixture containing the aqueous solutions of PLE and AgNO_3_ in 1:3 ratios (*v/v*), from pale yellow to light brown indicated the PLE bio-actives meditated bio-reduction of Ag^+^ to LIV-AgNPs after 20 min at 25 ± 5 °C. The color of reaction mixture tuned into intense brown after 24 h. The appearance of a sharp UV-Vis band at λ_max_428 nm was observed which is likely due to the surface plasmon resonance (SPR) of nascent LIV-AgNPs in colloidal solution (Figure 1a). The UV-Vis absorption peak position (400–500 nm) and formation of characteristic brown color LIV-AgNPs were found concordant with the reports published on plant mediated green synthesis of AgNPs [6]. Besides, UV-Vis absorption (λ_max_428 nm) analysis of colloidal LIV-AgNPs up to six months revealed that the NPs were highly stable as the experiments showed no significant change in SPR peak (Figure 1b).

#### 2.1.2. Assessment of Bio-Actives in Pristine PLE and LIV-AgNPs by GC-MS and FTIR

**Figure 2:**
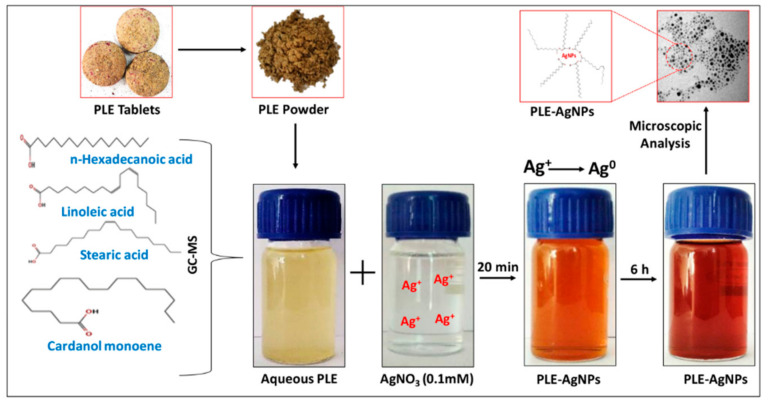
Schematic representation of LIV-AgNPs synthesis.

**Figure 3:**
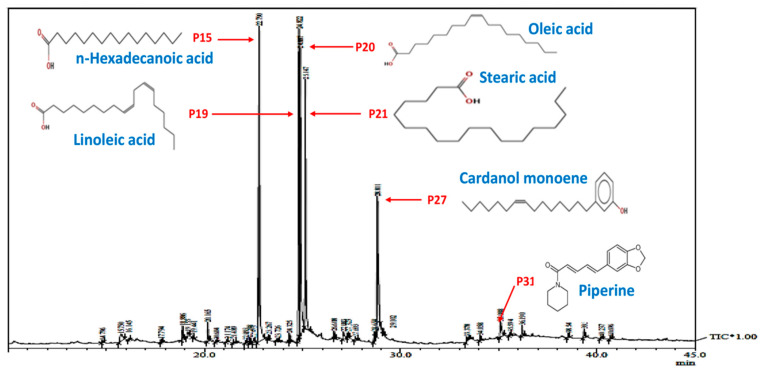
Gas chromatography mass-spectroscopic analysis. Typical GC–MS chromatogram of PLE showing a total 37 peaks (P) for different bio-actives compounds including six major compounds as n-hexadecanoic acid (P15—21.95%), linoleic acid (P19—20.45%), oleic acid (P20—18.01%), stearic acid (P21—13.99%), cardanol monoene (P27—11.92%) and piperine (P31—1.83%).

**Figure 4:**
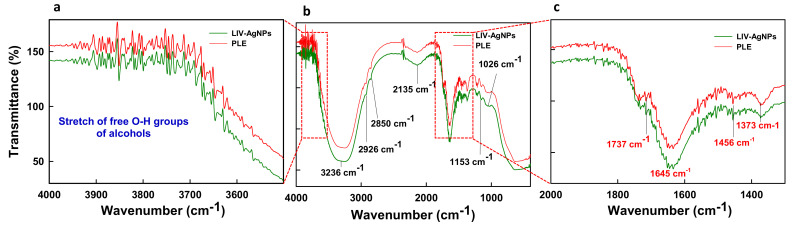
FTIR analysis of LIV-AgNPs. Panel (**a**,**c**) demonstrate feeble stretching signals of functional groups linked with bio-actives on PLE-alone (red) and LIV-AgNPs (green) in the range of 4000 to 3500 cm^−1^ and 2000 to 15,000 cm^−1^, at high magnification, respectively. Whereas, panel (**b**) represents a comparative FTIR spectra analysis of PLE-alone and LIV-AgNPs at full scan scale at normal magnification.

Before, synthesis of LIV-AgNPs, the pristine PLE was put through to GC-MS analysis [24] in order to presume plausible bio-active compounds that may acted as, (i) reducing agent for free metal cations (Ag^+^ → Ag^0^), (ii) stabilizing agent while growth on nascent NPs in progress during nucleation phase and (iii) capping of fully grown or stabilized NPs as described in our previous study [24] was illustrated in schematic mechanism of LIV-AgNPs formation (Figure 2). The GC-MS spectrum of pristine PLE (Figure 3) reflected a total of 37 peaks (P) for a variety of bio-actives were described in our previous study [24]. Based on their peak area, four major bio-actives in PLE were found to be long/short chained hydrocarbon fatty acids containing terminal -OH and -COOH groups, viz. n-hexadecanoic acid (P15—21.95%), linoleic acid (P19—20.45%), oleic acid (P20—18.01%) and stearic acid (P21—13.99%) [24]. Besides, two polyphenolic bio-actives were also detected namely cardanol monoene (P27—11.92%) and piperine (P31—1.83%) [24] likely play axillary role in bio-reduction and capping of NPs (Table S1) [24]. Next, the FTIR-based assessment of as-prepared LIV-AgNPs also demonstrated the presence of PLE bio-actives that can be argued being responsible for bio-reduction of metal cations into nascent NPs, stabilization and capping of AgNPs. The FTIR spectrum in Figure 4a–c, demonstrates a variety of molecular signatures of PLE bio-actives adsorbed on AgNPs, which in fact appeared as sharp, broad, strong and weak signals pertaining to their band behavior such as stretching, banding and vibrations. In Figure 4a, a dense area of FTIR spectrum ranged between 3500 cm^−1^ and 3700 cm^−1^ indicated the presence a majority of PLE bio-actives associated to AgNPs surface and hence we analyzed this area at a high resolution. The observations of this section suggest the presence of medium and sharp stretching were assigned to the free -OH groups of alcohols [25]. Whereas, strong and broad stretching around 3236 cm^−1^ confirmed the presence of intermolecular bonded -OH and -NH groups of carbohydrates/lipids and primary amines, respectively, as depicted in Figure 4b, signify the reduction of Ag^+^ to Ag^0^ and capping of AgNPs [26]. The weak vibrations between 2926 and 2850 cm^−1^, and 2135 cm^−1^ were assigned to stretching of C-H and C≡C groups of lipids and alkyne, respectively (Figure 4b). The peak at 1737 cm^−1^ is likely due to the presence of carbonyl (C-O) group of FAs, whereas peak at 1645 cm^−1^ represent carboxylic groups (C=O) of FAs and amine group (N-H) of protein (Figure 4c) [24,26]. Indeed, the appearance of C-O and C=O signals strongly advocate the involvement of FAs and proteins in bio-reduction and PLE bio-actives corona likely physisorbed on the surface of LIV-AgNPs. Besides, the peak at 1456 cm^−1^ can be ascribed to CH_2_ deformation or due to C–O–H bending, 1373 cm^−1^ represents O-H groups of phenolic compounds, signal at 1153 cm^−1^ was taken as C-O-C stretching which signified the presence of carbohydrates, peak around 1026 cm^−1^ was assigned to O-H stretching of polyphenols (Figure 4c) [26]). Overall, our GC-MS and FTIR results strongly suggest an active role of PLE attributed FAs and polyphenolic in the synthesis of LIV-AgNPs. In same line, Rao and Trivedi [27] have also demonstrated formation of FAs encapsulated AgNPs using stearic, palmitic and lauric acids as bio-reducing and stabilizing agents. Recently, the study of Gnanakani et al. [26] exhibited the FAs namely octadecanoic, hexadecanoic and octadecanoic acids in microalgae *Nannochloropsis* extract as potential bio-reducing and stabilizing agents in synthesis of AgNPs. Beyond the abundance of FAs, auxiliary phenolics, proteins, carbohydrates and enzymes bio-moieties in the benign milieu of PLE can be argued to play both key roles in plant extract mediated bio-fabrication of nanomaterials [25].

#### 2.1.3. Electron Microscopic Properties of LIV-AgNPs

**Figure 5:**
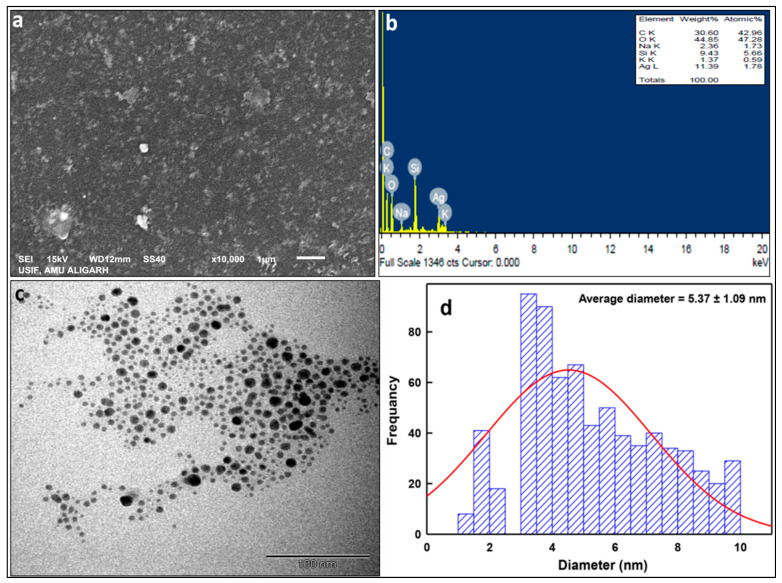
Electron microscopic analyses. Panel (**a**) shows SEM micrographs of LIV-AgNPs. Whereas, energy dispersive X-ray spectrum in panel (**b**) represents the percentage of Ag, C, O, NA, K and Si elements present in LIV-AgNPs. Panel (**c**) demonstrates TEM image of LIV-AgNPs whereas, panel (**d**) depicts the particle size distribution in TEM images, respectively.

The SEM micrographs in Figure 5a demonstrated a significant level of agglomerations in LIV-AgNPs when allowed to dry to solid powder. Besides, the elemental composition of PLE-AgNPs obtained by using EDS showed prominent peaks for carbon (30.6%), oxygen (44.85%) and silicon (9.43%) along with the characteristic peak of Ag (11.39%) at approximate 3 keV (Figure 5b). Contrarily to powdered LIV-AgNPs (Figure 5a), the TEM analysis of colloidal LIV-AgNPs solutions witnessed a great level of dispersity in aqueous environment, which was likely attributed by repulsion forces existed between two O-H groups hanging out from the soft PLE corona of AgNPs (Figure 5c). At the same time, the ImageJ software-based size determination on TEM micrographs revealed the sized of LIV-AgNPs was ranged between 1–10 nm with an average diameter of 5.37 ± 1.09 nm (Figure 5d).

#### 2.1.4. XRD Analysis of LIV-AgNPs

**Figure 6:**
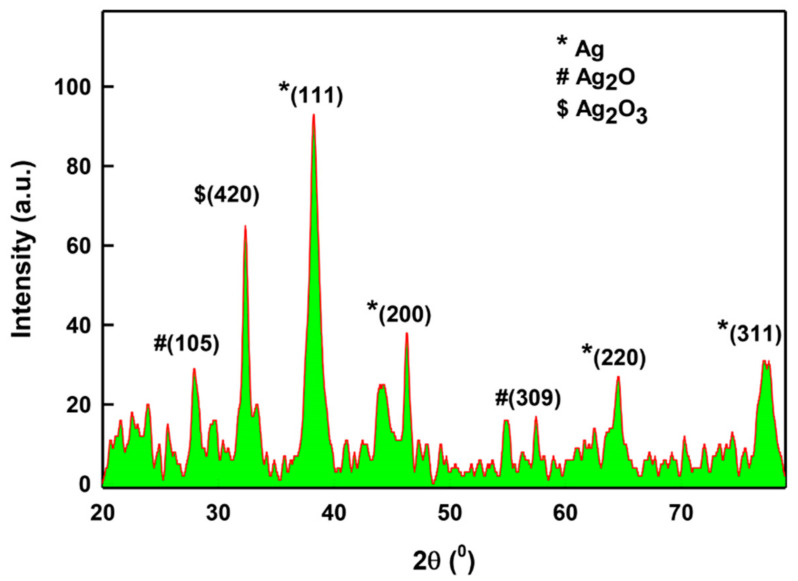
X-ray diffraction (XRD) pattern of LIV-AgNPs.

The structure and crystallite phase of LIV-AgNPs annealed at 60 °C was obtained by X-ray diffraction (XRD) at 2θ degree scale in the range between 20 and 80° (Figure 6). The main peaks (111), (200), (220) and (311) were observed at 38.3°, 46.2°, 64.5° and 78.2° respectively, suggest that as-prepared LIV-AgNPs are crystalline in nature [28,29]. In addition, the three peaks at 28° (105) and 57° (309), and 32.5° (420) indicated some traces of hexagonal Ag_2_O and orthorhombic Ag_2_O_3_ NPs, respectively [30]. Besides, the XRD also demonstrated feeble and broad reflections as noise, which might be due to the presence of bioorganic compounds on the surface of AgNPs [29]. The average particle size of LIV-AgNPs was estimated to be 8.53 nm, based on full-width-at-half-maximum (FWHM) value for (111) plane of reflection. The size of the as-synthesized NPs was found in agreement to size calculated by TEM analysis (5.37 ± 1.09 nm).

#### 2.1.5. Antibacterial and Anticandidal Activity of LIV-AgNPs

##### Growth Inhibition Activity Assessment and Minimal Inhibitory Concentration (MIC), Minimal Bactericidal Concentration (MBC) and Minimal Fungicidal Concentration (MFC) Values Determination

**Figure 7:**
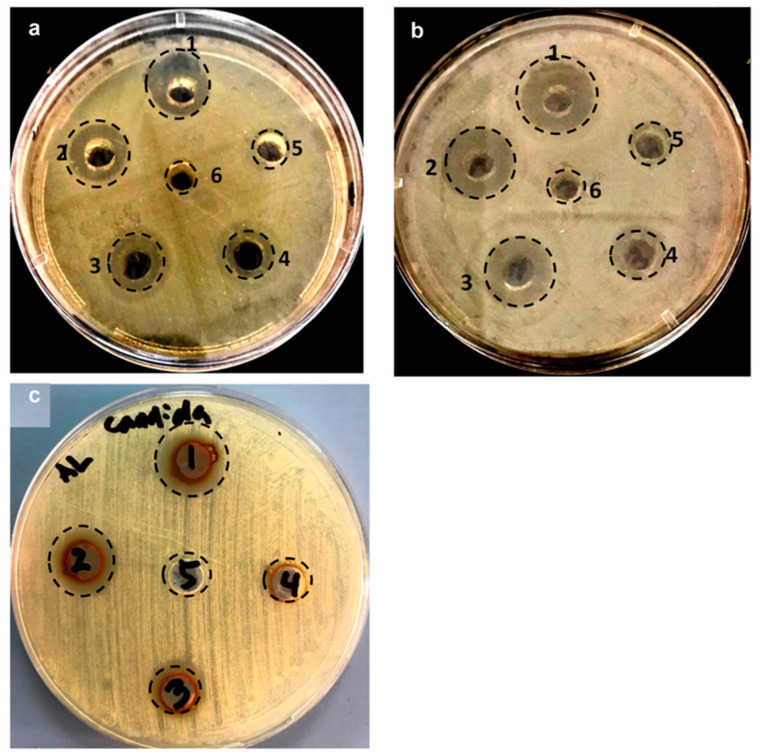
Assessment of antimicrobial activity of PLE alone and LIV-AgNPs by well diffusion assay. Comparative antimicrobial effects induced by, 100 μL of pristine PLE (well 6) as control and increasing amounts i.e., (well 5) 12.5, (well 4) 25, (well 3) 50, (well 2) 100 and (well 1) 200 μg/mL of LIV-AgNPs against the Gram-negative multiple drugs resistance *Pseudomonas aeruginosa* (MDR-PA) (panel-(**a**)) and Gram-positive methicillin-resistant *Staphylococcus aureus* (MRSA) (panel-(**b**)). Panel-(**c**) showing zone of inhibition of *C. albicans* where well (5) represent control i.e., 100 μL of pristine PLE, well (4) filled with 25, well (3) 50, (well 2) 100 and (well 1) 200 μg/mL of LIV-AgNPs, respectively.

The antimicrobial activity of as prepared LIV-AgNPs (12.5–200 µg/mL) was assessed against the MDR Gram-negative MDR-PA and Gram-positive MRSA bacteria, and *C. albicans* fungal strain using well-diffusion assay. The images in Figure 7a–c demonstrate a clear-cut antibacterial and antifungal activity of LIV-AgNPs against the test strains. We have observed an apparent increase in zone of inhibition (ZOI) against MDR-PA as 10.0 ± 2.2, 13.0 ± 2.0, 13.5 ± 1.5, 16 ± 2.0 and 18.5 ± 1.0 mm at 12.5, 25, 50, 100 and 200 µg/mL of LIV-AgNPs, respectively (Figure 7a, Table 1). Whereas, the increase in diameter of ZOI in Gram-positive MRSA found to be 10.5 ± 2.5, 12 ± 0.5, 16.5 ± 1.5, 19.5 ± 4.5 and 22.5 ± 1.5 mm, respectively, compared to control wells (8.0 ± 1.2), under the identical conditions (Figure 7b, Table 1). In the case of *C. albicans* fungi, higher doses of LIV-AgNPs (50, 100 and 200 µg/mL) could exert antifungal effects as 13.75 ± 1.0, 15 ± 2.2 and 16.50 ± 1.2 mm, respectively (Figure 7c, Table 1). Overall, these dose-dependent trends in antimicrobial activities of LIV-AgNPs were strongly supported by earlier reports on antibacterial and antifungal activities of plant bio-actives capped NPs, published elsewhere [5,31]. Besides, the MICs values of LIV-AgNPs against MDR-PA, MRSA and *C. albicans* were determined as 258.3 ± 1 4.4, 516.8 ± 28.9 and 758.3 ± 38.2 µg/mL, respectively. The MBC values for MDR-PA and MRSA were 516.7 ± 28.9 and 1033.3 ± 57.7 µg/mL, respectively, while MFC value was found as 1533.3 ± 57.7 µg/mL against *C. albicans*.

##### SEM Based Analysis of LIV-AgNPs Interaction and Cellular Damage

**Figure 8:**
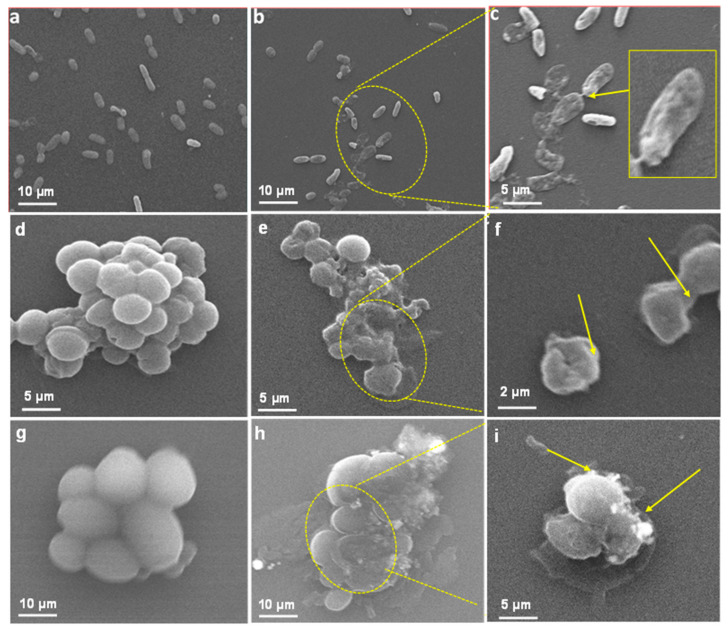
Interaction between LIV-AgNPs and microbial cells. Panels (**a**,**d**,**g**) represent SEM images of MDR-PA, MRSA and C. *albicans* cells in the absence of PLE-AgNPs. Whereas, panels (**b**,**c**,**e**–**i**), demonstrate cellular damage in MDR-PA, MRSA and C. *albicans* cells in presence of 100 µg/mL of LIV-AgNPs at low (10–5 µm) and high (5–2 µm) scales, respectively.

To validate the antibacterial and antifungal activities of LIV-AgNPs, the treated and untreated cells of test strains were compared under SEM visualization. The results in Figure 8b–c exhibited significantly ruptured cell wall with deep pits and cavities formation in MDR-PA cells treated with 100 µg/mL of LIV-AgNPs, which were likely due to internalization and surface contact killing or on-site augmented cations mediated toxicity, as described elsewhere [25]. Under identical conditions, Gram-positive MRSA cells were observed with significant structural damage along with tremendous bulging and deep cuts in cell membrane (Figure 8e,f), which indicated increased cytoplasmic granularity likely due to prompted interaction and internalization of LIV-AgNPs as compared to untreated cells (Figure 8d) [5]. Similarly, in the case of fungi, the LIV-AgNPs exposed *C. albicans* cells showed significant changes in native morphology such as deep pits in cells compared to untreated control (Figure 8h,i) as reported elsewhere [32]. Besides, Anuj et al. [33] have demonstrated a steady release of Ag^+^ from AgNPs and thus accumulated cations can destabilize cell membrane to combat with efflux-mediated drug resistance in Gram-negative bacteria. Recent study of Al-Kadmy [34] has also suggested that coating of AgNPs had enhanced penetrative ability through the cell wall and kills the *E. coli, S. aureus* and vancomycin resistant *Enteroccci* cells on banknote currency effectively under tentative conditions, as compared to AgNO_3_.

##### Antibiofilm Studies of LIV-AgNPs

**Figure 9:**
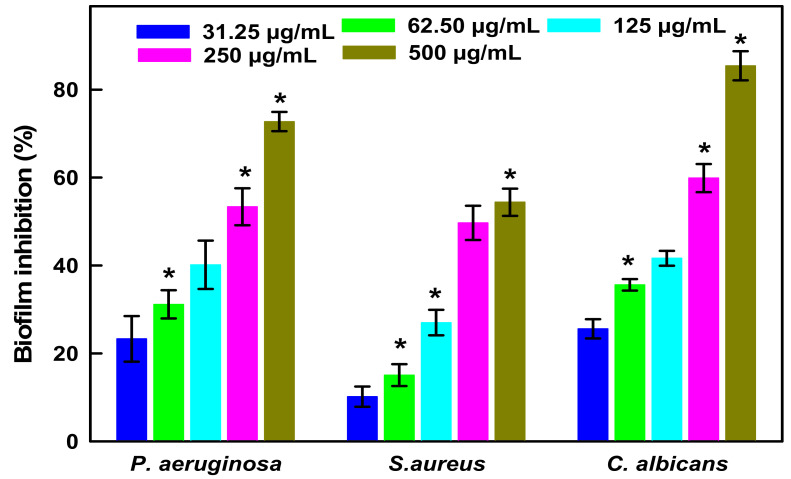
Antibiofilm activities. LIV-AgNPs concentration dependent inhibition in biofilm formation of MDR-PA, MRSA and *C. albicans* strains. The error bars represent mean ± S.D. of two independent experiments done in triplicates. * *p* < 0.05 versus control.

**Figure 10:**
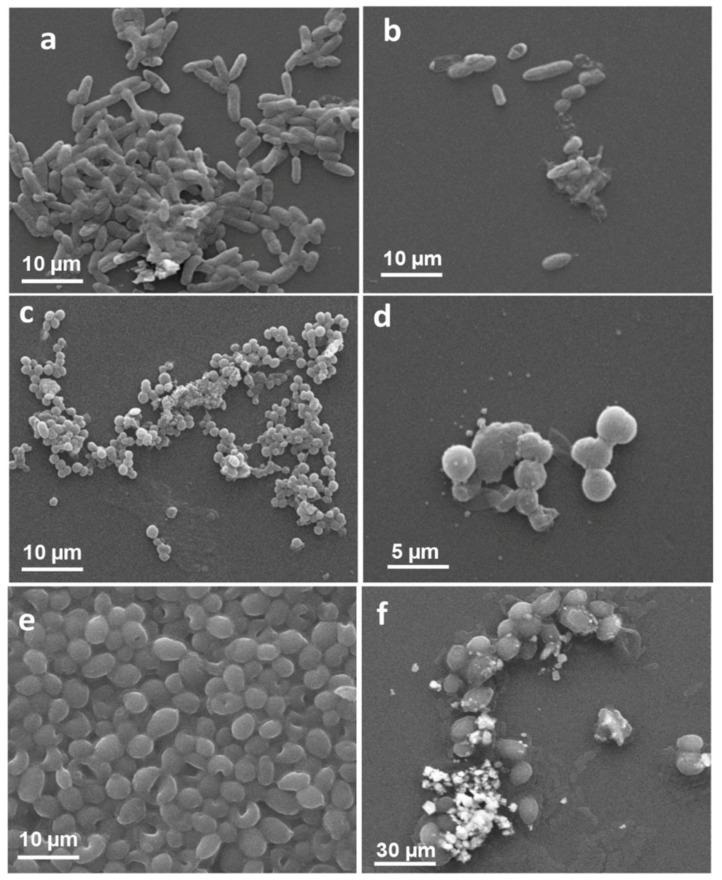
Microscopic analysis of biofilm inhibition. SEM micrographs of biofilm formed by MDR-PA, MRSA and *C. albicans* strains grown in absence (**a**,**c**,**e**) and presence (**b**,**d**,**f**) of 100 µg/mL of LIV-AgNPs.

Both, bacterial cells; Gram-negative MDR-PA and Gram-positive MRSA, and *C. albicans* fungi are well known for their biofilm producing ability and chronic nosocomial infections spread in hospital and associated settings [35,36]. Although, several metallic nanoantibiotics were found having great potential either to cease or eradicate biofilm adherence [37]. Whereas, the propensity of nanoantibiotics to readily diffuse through the biofilm biomass in order to reach microbial cells seemed to be compromised due to enzymatic, non-enzymatic and pH mediated degradations [38]. Interestingly, the evidence suggests that FAs, either free or physisorbed on to surface of NPs can (i) suppress the regulation of quorum-sensing (QS) genes, (ii) quenched the diffusible QS signal factors such as acyl-homoserine lactones and autoinducer-2 (AI-2) and (iii) dysregulate the associated non-QS targets like efflux pumps, oxidative stress and ergosterol synthesis [39,40,41]. Taken together the antimicrobial potential of FAs and AgNPs, we tested LIV-AgNPs for their antibiofilm activities. In, fact, our GC-MS results prompted us to consider the LIV-AgNPs as encapsulated by PLE bio-active FAs viz. n-hexadecanoic acid (P15—21.95%), linoleic acid (P19—20.45%), oleic acid (P20—18.01%) and stearic acid (P21—13.99%) (Figure 3, Table S1) [24] and hence responsible for significant anti-biofilm activities against MDR-PA, MRSA and *C. albicans*. The data in Figure 9 revealed the inhibition of biofilm formation by MDR-PA cells as 23.31 ± 5.2%, 31.17 ± 3.2%, 40.16 ± 5.5%, 53.37 ± 4.2% and 72.75 ± 2.2%, at 31.25, 62.50, 125, 250 and 500 µg/mL of LIV-AgNPs, respectively, versus untreated control (100%). Under identical conditions, MRSA cells could limit the accumulate biofilm mass as 10.17 ± 2.3%, 15.06 ± 2.5%, 27.00 ± 2.9%, 49.70 ± 3.9% and 54.40 ± 3.1%, respectively. Besides bacterial cells, the biofilm formed by *C. albicans* was also found declined significantly (*p* < 0.05 *) as 25.60 ± 2.2%, 35.60 ± 1.3%, 41.65 ± 1.7%, 59.9 ± 3.2% and 85.44 ± 3.3%, respectively. In parallel, the SEM based comparative analyses of untreated controls (Figure 10a,c,e) and LIV-AgNPs (100 µg/mL) treated MDR-PA (Figure 10b), MRSA (Figure 10d) and *C. albicans* (Figure 10f) cells were resulted in significant disruption in their biofilm architectures. Overall, the obtained trends in biofilm formation suggest that FAs hold a great potential to inhibit or disrupt biofilm formation against several microbial pathogens, including *S. aureus* [42], *P. aeruginosa* [43] and *C. albicans* [39,44]. Beyond the proven antibacterial and antibiofilm track record of AgNPs [45,46,47], a variety of FAs have earlier been warranted as potential antimicrobial agent. For instance, study of Santhakumari et al. [48] demonstrated hexadecanoic acid (100 µg/mL) could interrupted the QS by loosening of biofilm architecture (>60%) of *vibrios* spp. like *Vibrio harveyi*, *V. parahaemolyticus*, *V. vulnificus* and *V. alginolyticus* without affecting their planktonic growth. Besides, 12.8 µg/mL of hexadecanoic acid alone could inhibit the biofilm formation in *P. aeruginosa* and *E.coli* as 64% and 81%, respectively [43]. In the same context, Soni et al. [49] also demonstrated that palmitic acid (hexadecanoic acid), stearic acid, oleic acid and linoleic acid present in extract of ground beef inhibit the auto-inducer signals activity of the reporter strain (*Vibrio harveyi*) and reduced *E. coli* biofilm formation.

### 2.2. Antiproliferative Properties of LIV-AgNPs on Human Colon Cancer Cells (HCT-116)

#### Cell Viability Assay by MTT and Microscopic Analysis of HCT-116 Cells

**Figure 11:**
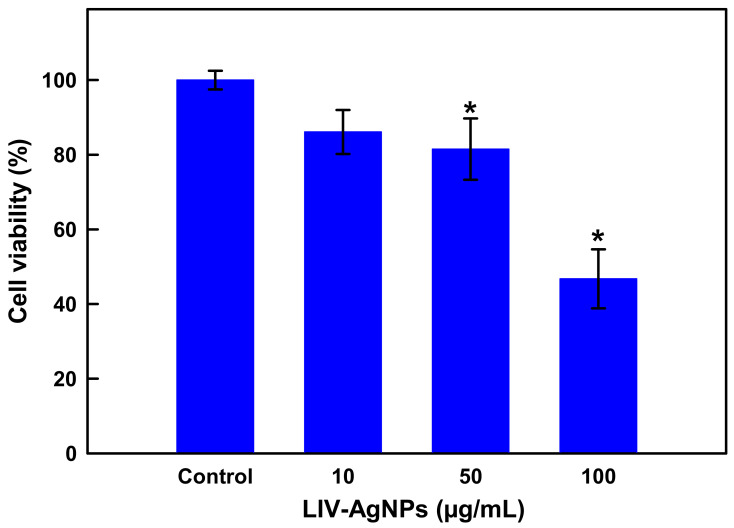
Dose-dependent cytotoxic effects of LIV-AgNPs against human colon cancer (HCT-116) cells quantitated by MTT assay after 24 h of incubation. Histograms are the mean ± S.D. of three independent experiments done in triplicate. * *p* < 0.05 verses untreated control.

**Figure 12:**
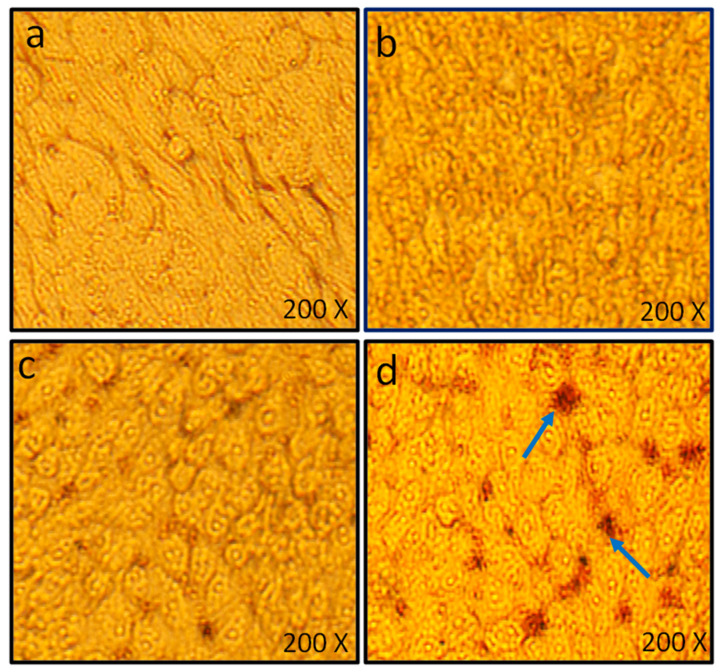
Microscopic analysis of HCT-116 cells exposed to varying concentrations of LIV-AgNPs for 24 h. Cytotoxic effects were manifested as shrunken morphology, gaps between the neighboring cells and cellular detachments, which appeared as round bodies in the culture medium. Images were captured at the magnification of 200X using a bright field inverted microscope (Olympus, CKX41, Tokyo, Japan). (**a**) HCT-116 untreated cells, (**b**–**d**) HCT-116 cells treated with—10, 50 and 100 µg/mL of LIV-AgNPs, respectively.

In addition to antimicrobial activities, PLE-capped AgNPs were also assessed for their anticancer potential. For this, human colon cancer cells were-cultured with colloidal LIV-AgNPs (10–100 µg/mL) for 24 and the nano-toxicity of LIV-AgNPs against HCT-116 cells was measured by employing colorimetric MTT assay. Precisely, compared untreated control cells (100 ± 2.5%), there is an apparent decline trend in cell viability as 86.10 ± 5.9%, 81.5 ± 8.2% and 46.75 ± 7.9% at 10, 50 and 100 µg/mL of LIV-AgNPs, respectively (Figure 11). At about 100 μg/mL, we observed a ca. 50% inhibition of the cell proliferation after 24 h. In parallel, HCT-116 cells exposed to LIV-AgNPs (10, 50 and 100 µg/mL) were also investigated for NPs induced morphological changes. The representative micrographs of HCT-116 cells clearly demonstrate that treatment of LIV-AgNPs caused significant morphological changes (Figure 12 b–d) as compared to untreated cells (Figure 12a). Our results were strongly supported by the findings of Kuppusamy et al. [50] who determined the IC_50_ value of their *Commelina nudiflora* capped-AgNPs as 100 µg/mL against cultured HCT-116 after 24 h. Besides, as compared to a single extract like *Chlorophytum borivilianum* extract functionalized AgNPs, which showed IC_50_ value of 254 μg/mL [51], the as prepared poly-herbal encapsulated LIV-AgNPs can act as much effective anticancer nanomedicine against human colon cancer cells. In this context, linolenic acid polymers impregnated to AgNPs have also been reported to show 82.3% inhibition rate against the rat pheochromocytoma PC 12 tumor cell line [52]. Similarly, fatty acids rich *Argemone mexicana* extract encapsulated AgNPs (100 µg/mL) were found to inhibit 80% human cervical cancer cell line (SiHa) proliferation [53]. The AgNPs have also been reported disrupting respiratory chain and cell division while releasing Ag+ in order to augment enhanced bacterial killing. It has reported that coating of AgNPs can result in improved functionality and corrosion resistance of magnesium structures in biomedical settings [54]. With the widespread application and inevitable environmental exposure, AgNPs can be accumulated in various organs. More serious concerns are raised on the biological safety and potential toxicity of AgNPs in the central nervous system (CNS), especially in the hippocampus. Further, Chang et al. [54] investigated the biological effects and the role of PI3K/AKT/mTOR signaling pathway in AgNPs mediated cytotoxicity using the mouse hippocampal neuronal cell line (HT22 cells). They found that AgNPs reduced cell viability and induced membrane leakage in a dose-dependent manner and AgNPs also promoted the excessive production of reactive oxygen species (ROS) and caused the oxidative stress in HT22 cells [54].

## 3. Materials and Methods

### 3.1. Preparations of Aqueous Extract of Liv52 Drug

To prepare the fatty acids rich poly-herbal Liv52 drug extract, Liv52 tablets (Himalaya Global Holdings Ltd., Bangalore, India), were crushed to fine powder and 5 g was then dissolved in 100 mL of ultra-pure water. After 1 h, the PLE solution was centrifuged at 12,000 rpm for 10 min and so collected supernatant was additionally filtered through the Wattman paper No. 1 [24]. Thus, obtained aqueous PLE was stored at 4 °C for the green synthesis of LIV-AgNPs.

### 3.2. GC-MS Based Assessment of Bio-Actives in Poly-Herbal Liv52 Drug Extract (PLE)

Considering the fact that Liv52 is a poly-herbal composition of *C. spinosa, C. intybus, S. nigrum, T. arjuna* and *A. millefolium* extracts [23], the gas chromatography mass-spectroscopy (GC-MS) based analysis on methanolic extract of PLE was performed to ascertain the bio-actives compounds that plausible involved in reduction, capping and stabilization of LIV-AgNPs, following the method described elsewhere [24,31].

### 3.3. Nanofabrication of Poly-Herbal liv52 Drug Extract Capped AgNPs (LIV-AgNPs)

For the synthesis of LIV-AgNPs, PLE (25 mL) was mixed into 75 mL of 0.1 mM AgNO_3_ solution. The reaction mixture was then kept in dark at room temperature (30 ± 5 °C). The color of reaction mixture was changed from pale yellow to brown after 20 min and became even dark brown within 24 h, which indeed indicated the reduction of Ag^+^ to Ag^0^ NPs [8].

### 3.4. Characterization of Synthesized LIV-AgNPs

#### 3.4.1. UV–Vis Spectroscopy and FTIR Analysis

Formation of LIV-AgNPs was monitored by using UV-Vis spectroscopy in range of 300–800 nm as described recently elsewhere [55]. The Fourier-transform infrared spectroscopy (FTIR) was performed to ascertain the presence of PLE bio-actives that have likely played either key or auxiliary role in the reduction Ag^+^ to Ag^0^, stabilization of nano silver and capping of nascent LIV-AgNPs during synthesis [8].

#### 3.4.2. Electron Microscopic and EDS Analysis of LIV-AgNPs

The shape, size and elemental composition of LIV-mediated synthesized AgNPs was carried out by scanning electron microscope (SEM), transmission electron microscope (TEM) and energy dispersive spectroscopy (EDS) following the methods described in our previous study [56].

#### 3.4.3. XRD Analysis of LIV-AgNPs

The crystallinity and size of bio-synthesized LIV-AgNPs was analyzed by XRD machine as protocol described recently [57].

### 3.5. Microbial and Human Carcinoma Cell Cultures

In this study, multi-drug resistant *Pseudomonas aeruginosa* (laboratory strain), methicillin-resistant *Staphylococcus aureus* (ATCC 33591) and *Candida albicans* (ATCC 14053) were used to investigate the antibacterial, anticandidal and antibiofilm activities of synthesized PLE-AgNPs. For anticancer efficacy assessment, the human colon cancer (ATCC No. CCL-247) cell line was used. Both, the microbial and human carcinoma cell cultures were maintained as described in earlier studies [9,58].

### 3.6. Assessment of Antimicrobial Efficacy of LIV-AgNPs

#### 3.6.1. Minimal Inhibitory Concentration, Minimal Bactericidal Concentration, Minimal Fungicidal Concentration and Zone of Inhibition Determination

The antibacterial and antifungal activity of synthesized LIV-AgNPs was carried out using two-fold micro broth dilution method in the range of 62.5 to 2000 µg/mL against Gram-negative MDR-PA, Gram-positive MRSA and *C. albicans* fungal strains as method described by Ansari et al. [59]. The MIC value is defined as the lowest concentration of LIV-AgNPs at which no visible growth of bacteria and Candida was observed. After MIC determination of LIV-AgNPs, aliquots of 100 µl from wells having no visible growth was seen were further spread on MHA and SDA plates for 24 h at 37 °C and 28 °C, respectively, to calculate the MBC and MFC values. The lowest concentration of LIV-AgNPs that kills 100% population of tested bacteria and Candida, is considered as MBC/MFC values [59].

Further, agar well diffusion assay was performed to determine the zone of inhibition (in millimeter) of LIV-AgNPs against Gram-negative MDR-PA, Gram-positive MRSA and *C. albicans* as method described by Jalal et al. [8].

#### 3.6.2. Ultrastructural Alteration Caused by LIV-AgNPs in Bacterial and Candidal Cells

The morphological changes caused by LIV-AgNPs in bacterial and yeast strains cells were examined by SEM analysis following protocol described in previous reports [60]. Briefly, ~10^6^ CFU/mL of MDR-PA, MRSA, and *C. albicans* cells treated with 100 µg/mL of LIV-AgNPs were incubated at 16 h at a recommended temperature. Thereafter, washing of treated and untreated samples were performed using centrifugation and then the pellets was fixed with glutaraldehyde (4% *v/v*) followed by osmium tetroxide (1%). After fixations, dehydration, drying and gold coating was performed and finally the effects of LIV-AgNPs on test strains of bacteria and *Candida* was seen under SEM at an accelerated voltage of 20 EV [61].

#### 3.6.3. Inhibition of Biofilm Forming Abilities of MDR-PA, MRSA and C. albicans

The inhibition in biofilm formation after treatment with LIV-AgNPs was quantitated by employing the microtiter crystal violet assay [61]. Briefly, 20 µl of freshly cultured MDR-PA, MRSA and *C. albicans* were admixed with 180 µl of varying concentrations (31.25, 62.50, 125, 250 and 500 µg/mL) of as prepared LIV-AgNPs and then the plates were kept in incubator for 24 h. The cells without LIV-AgNPs were considered as control group. After incubation, the content from the microtiter wells were decanted and gently washed with PBS and left for drying. The adhered biofilm biomass was then stained with crystal violet solution (0.1% *w/v*) for 30 min. The excess dyes were decanted and washed again with PBS and dried the wells completely. So stained biofilm was then solubilized with 95% ethyl alcohol and quantitated by optical density at 595 nm [62].

#### 3.6.4. Visualization of Biofilm Architecture by SEM

Besides, the effect of LIV-AgNPs on MDR-PA, MRSA and *C. albicans* biofilm architecture was investigated by SEM [62]. In brief, 100 µl fresh cultures of tested bacterial and yeast strains with and without LIV-AgNPs were inoculated on a glass coverslip in a 12-wells plate for overnight. After incubation, the glass coverslips were taken off and washed with PBS to remove the unadhered cells. After washing, the coverslips were fixed with glutaraldehyde (2.5% *v/v*) for 24 h at 4 °C. After fixation, washed the coverslips again and then subjected it to dehydration, drying and gold coating. After that, the effects of LIV-AgNPs on biofilm of tested bacteria and yeast were observed using SEM [61].

### 3.7. Evaluation of Anticancer Potential of LIV-AgNPs

#### 3.7.1. MTT Assay

Human colorectal carcinoma cell line was used to investigate the anticancer potential of synthesized LIV-AgNPs at different concentrations (10, 50 and 100 µg/mL) in a 96-well cell culture plates by measuring optical density at 570 nm and the cell viability (%) was estimated using given formula [62].

$$\%\;\mathrm{of}\;\mathrm{cell}\;\mathrm{viability}=\frac{\mathrm{Optical}\;\mathrm{density}\;\mathrm{of}\;\mathrm{NPs}-\mathrm{treated}\;\mathrm{cells}\;}{\mathrm{Optical}\;\mathrm{density}\;\mathrm{of}\;\mathrm{control}\;\mathrm{cells}}\times 100$$

#### 3.7.2. Effects of LIV-AgNPs on Morphology of HCT-116 Cells

The effect of different concentrations (10, 50 and 100 µg/mL) of LIV-AgNPs was validated by subsequent SEM analysis of HCT-116 cells at the end of experiments under an inverted microscope equipped with a digital camera [62].

### 3.8. Statistical Analysis

Statistical analysis of data was done by one-way analysis of variance (ANOVA), Holm–Sidak method, multiple comparisons versus the control group (Sigma Plot 11.0, San Jose, CA, USA). The results indicate mean ± S.D. values determined with three independent experiments done in triplicate. The level of statistical significance chosen was * *p* < 0.05 unless otherwise stated.

## 4. Conclusions

This study demonstrates a simple one-pot procedure for synthesis of fatty acids rich aqueous extract of poly-herbal drug Liv52 stabilized LIV-AgNPs. GC-MS results demonstrated substantial proofs that PLE contributed terminal –OH and –COOH functional groups bearing FAs, namely n-hexadecanoic acid (21.95%), linoleic acid (20.45%), oleic acid (18.01%) and stearic acid (13.99%), that were speculated to reduce Ag^+^ into Ag^0^ and followed by stabilization with soft corona formation around the nascent NPs surface during synthesis reaction. Besides, the LIV-AgNPs were found to be potential nano-therapeutics agents in order to control bacterial growth and biofilm formation against Gram-negative MDR-PA, Gram-positive MRSA and *C. albicans* strains, in vitro. Significant interaction of PLE-AgNPs with both, Gram-negative and Gram–positive bacterial and fungal strains was observed. The propensity of LIV-AgNPs interaction and internalization in planktonic cells as well as biofilm biomass appeared clearly in SEM analysis of treated experimental sets of MDR-PA, MRSA and *C. albicans* owing to the difference in their cell wall composition. However, the antibacterial and antibiofilm potential of LIV-AgNPs might be due to a swift surface contact through a stubborn biofilm matrix formed around the colonized cells requires further investigations to understand the mechanism of their action mode for nanoantibiotics development. In addition, the dose-dependent cytotoxicity trend of LIV-AgNPs against cultured human colon cancer cells ensured that the FAs-rich PLE capped nanomaterials could act as potential anticancer nanodrugs. However, the anticancer data of LIV-AgNPs here reported are only preliminary and will be successively deeply investigated exploring their cytotoxicity on normal cells as well as the antiproliferative activity of LIV-52 extract alone, as control.

## Figures and Tables

**Table 1:** Determination of zone of inhibition (in mm) values of LIV-AgNPs.

Well No.	LIV-AgNPs (µg/mL)	Diameter of Growth Inhibition Zone (mm)		
		MDR-PA	MRSA	*C. albicans*
1	200	18.5 ± 1.0	22.5 ± 1.5	16.5 ± 1.2
2	100	16.0 ± 2.0	19.5 ± 4.5	15.0 ± 2.2
3	50	13.5 ± 1.5	16.5 ± 1.5	13.7 ± 1.0
4	25	13.0 ± 2.0	12.0 ± 0.5	ND
5	12.5	10.0 ± 2.2	10.5 ± 2.5	ND
6	Control (PLE)	ND	ND	ND

ND = not detected.

## Data Availability

The data presented in this study are available in this manuscript.

## Supplementary Materials

The following are available online at https://www.mdpi.com/1424-8247/14/2/139/s1, Table S1: GS-MS analysis of poly-herbal drug Liv52 extract (PLE).
