# Supplementary material for: Biofabricated Fatty Acids-Capped Silver Nanoparticles as Potential Antibacterial, Antifungal, Antibiofilm and Anticancer Agents
Source: Pharmaceuticals (Basel). 2021 Feb 9;14(2):139. doi: 10.3390/ph14020139 (PMC7915658; doi:10.3390/ph14020139)
Supplement: Supplementary file 1 [file pharmaceuticals-14-00139-s001.pdf]

Table. S1. GS-MS analysis of poly-herbal drug Liv52 extract (PLE).

Peak (P)	Retention Time	Peak Area (%)	Name of compounds	Mol. formula	Mol. Weight	Structure
1	14.796	0.16	Octanoic Acid, 3-Oxo-, Methyl Ester	$C_9H_{16}O_3$	172	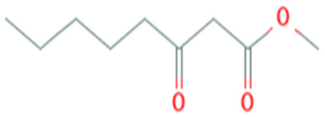
2	15.750	1.30	1,3-Propanediol, 2-(hydroxymethyl)-2-nitro-	$C_4H_9NO_5$	151	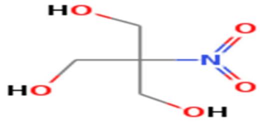
3	16.145	0.11	Benzene, 1-(1,5-Dimethyl-4-Hexenyl)-4-Methyl-	$C_{15}H_{22}$	202	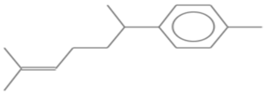
4	17.794	0.17	1-Tridecanol	$C_{13}H_{28}O$	200	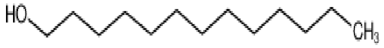
5	18.886	0.86	Ar-tumerone	$C_{15}H_{20}O$	216	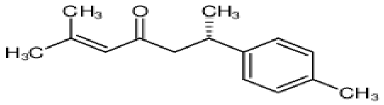
6	19.155	0.55	Mome Inositol; (methyl α,β-D-glucofuranose)	$C_7H_{14}O_6$	194	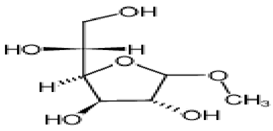

7	19.441	0.25	Alpha.-tumerone	$C_{15}H_{22}O$	218	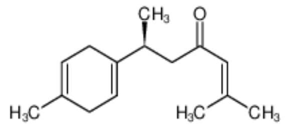
8	20.165	1.14	Tetradecanoic acid	$C_{14}H_{28}O_2$	228	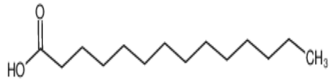
9	20.604	0.10	1-Tetradecanol	$C_{14}H_{30}O$	214	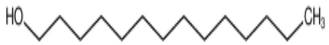
10	21.174	0.10	Neophytadiene	$C_{20}H_{38}$	278	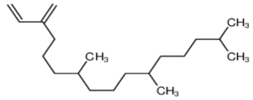
11	21.489	0.19	Didodecyl phthalate	$C_{32}H_{54}O_4$	502	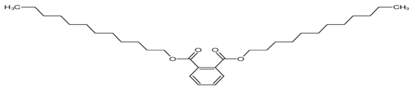
12	22.093	0.09	7,9-Di-tert-butyl-1-oxaspiro(4,5)deca-6,9-diene-2,8-dione	$C_{17}H_{24}O_3$	276	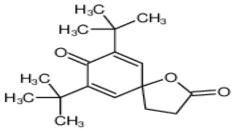
13	22.298	0.25	Hexadecanoic acid, Methyl Ester	$C_{17}H_{34}O_2$	270	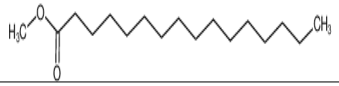
14	22.493	0.33	9-Eicosyne	$C_{20}H_{38}$	278	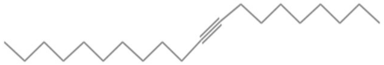
15	22.790	21.95	n-Hexadecanoic acid (Palmitic acid)	$C_{16}H_{32}O_2$	256	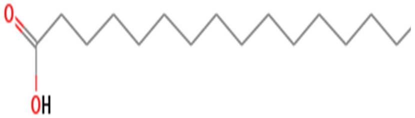

16	23.267	0.25	Azuleno [4,5-b] furan-2 (3H)-one,	$C_{15}H_{18}O_2$	230	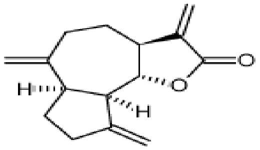
17	23.726	0.25	Palmitic Acid, TMS derivative	$C_{19}H_{40}O_2Si$	328	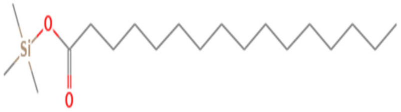
18	24.325	0.32	9,12-Octadecadienoic acid, methyl ester	$C_{19}H_{34}O_2$	294	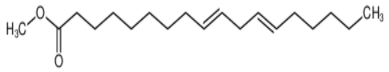
19	24.822	20.45	9,12-Octadecadienoic acid (Z,Z)- (Linoleic Acid)	$C_{18}H_{32}O_2$	280	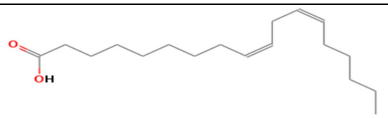
20	24.887	18.01	9-Octadecenoic acid (Z)- (Oleic acid)	$C_{18}H_{34}O_2$	282	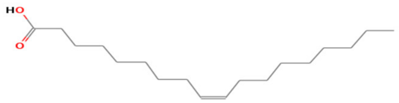
21	25.147	13.99	Octadecanoic acid (Stearic acid)	$C_{18}H_{36}O_2$	284	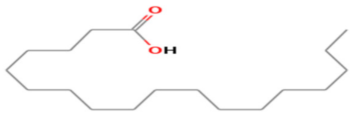
22	26.608	0.45	1-(4-Hydroxy-3-methoxyphenyl)dec-4-en-3-one	$C_{17}H_{24}O_3$	276	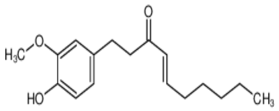
23	27.083	0.73	Z,Z-8,10-Hexadecadien-1-ol	$C_{16}H_{30}O$	238	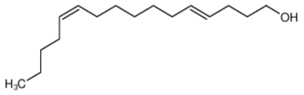

24	27.323	0.42	9-Octadecenoic acid (Z)-	$C_{18}H_{34}O_2$	282	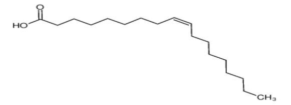
25	27.693	0.29	Phosphonic acid, dioctadecyl ester	$C_{36}H_{75}O_3P$	586	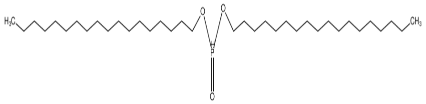
26	28.658	0.42	Ginkgol (TMS)	$C_{24}H_{42}OSi$	374	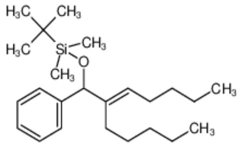
27	28.811	11.92	(Z)-3-(pentadec-8-en-1-yl)phenol (Cardanol monoene)	$C_{21}H_{34}O$	302	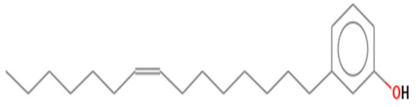
28	29.102	0.17	Phenol, 3-pentadecyl-	$C_{21}H_{36}O$	304	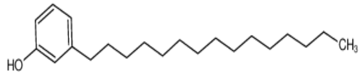
29	33.378	0.39	Piperine	$C_{17}H_{19}NO_3$	285	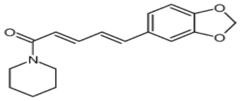
30	34.058	0.26	Squalene	$C_{30}H_{50}$	410	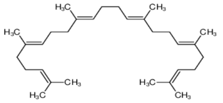

31	35.088	1.83	Piperine	$C_{17}H_{19}NO_3$	285	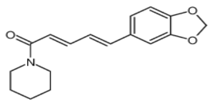
32	35.594	0.16	(2E,4E,10E)-N-Isobutylhexadeca-2,4,10-trienamide	$C_{20}H_{35}NO$	305	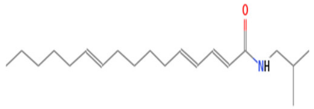
33	36.190	0.66	Doconexent, TBDMS derivative	$C_{28}H_{46}O_2Si$	442	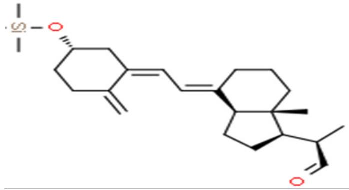
34	38.547	0.34	Stigmasta-5,22-dien-3-ol, (3.beta.,22E)-	$C_{29}H_{48}O$	412	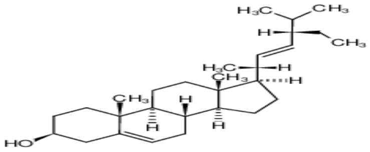
35	39.357	0.72	Stigmast-5-en-3-ol, (3.beta.)-	$C_{29}H_{50}O$	414	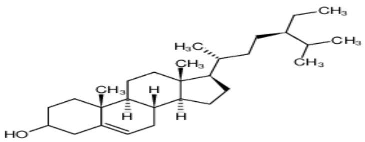
36	40.237	0.19	2-Hydroxymethyl-2,6,8,8-tetramethyltricyclo[5.2.2.0(1,6)]undecane	$C_{16}H_{28}O$	236	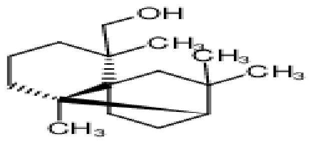

37	40.696	0.25	Lupeol	$C_{30}H_{50}O$	426	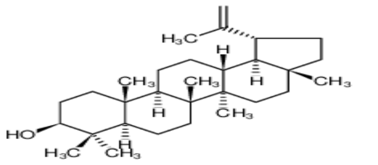
		**100**				
